# Review of Hematopathology Consult Cases: A Two-Year Experience in a Tertiary Referral Center in Lebanon

**DOI:** 10.1155/2018/3028625

**Published:** 2018-12-30

**Authors:** Sara A. J. Sinno, Zaher I. Chakhachiro, Samer R. Nassif

**Affiliations:** Department of Pathology and Laboratory Medicine, American University of Beirut Medical Center, Beirut, Lebanon

## Abstract

Hematopathology remains a difficult diagnostic field. With the significant ongoing changes in the classification system that happened over the past several decades, the general pathologist faces many challenges when dealing with patients suspected to have lymphoma or leukemia. The authors assessed referred hematopathology cases that were reviewed by specialized hematopathologists. Of 309 cases, major discrepancy was found in 23% of them. The discrepancy ranged from lymphoma reclassification to other major revisions that had significant impact on patient treatment and management. This paper highlights some of the challenges that may face the general practicing pathologist when dealing with suspected hematopoietic neoplasms.

## 1. Introduction

During the past several decades, many classification systems for hematopoietic neoplasms have been proposed. Examples include the Rappaport, Lukes-Collins, and Kiel classification systems and the Working Formulation [1]. Such classification systems resulted in high rates of diagnostic discrepancies among pathologists [1–6]. The introduction of the World Health Organization (WHO) classification in 2000, followed by its 2008 and 2016 updates, established a more unified classification system among pathologists and emphasized the importance of integrating clinical, morphological, immunophenotypic, and genetic information in reaching the proper diagnosis [7–9]. Nonetheless, there remained a significant amount of diagnostic discrepancies observed in cases sent for hematopathology expert reviews [1, 7, 10–16], and previous studies have shown widely variable discordance rates ranging from 6% to 55% [1, 13]. However, to date, no such studies have been conducted in the Middle East world, a region that may sometimes lack some of the more advanced technologies.

Frequently, hematopathology referral cases from within Lebanon and from other Arab countries are received at the pathology department at the American University of Beirut Medical Center (AUB-MC) for review, either for confirmation of diagnoses rendered in other institutions or to issue primary diagnoses on specimen collected in other centers. Commonly, examination of such specimen requires ancillary testing such as immunohistochemical staining, in situ hybridization staining, or molecular studies, some of which are tests that may not be available at the referring institution or laboratory. While some of the outside cases are referred with preliminary diagnoses pending ancillary studies, there is a significant portion of cases received with a rendered final diagnosis. Of these cases, there is a subset where a major diagnostic discrepancy was found, something that greatly affected the optimal course of patient treatment, particularly when patients had already been treated or started treatment prior to presenting to AUB-MC. Therefore, in order to determine the rate of diagnostic discrepancies and to identify the specific limiting factors and difficulties that pathologists in Lebanon and in the Arab world face precluding rendering adequate diagnoses, we reviewed all the hematopathology cases that were sent for referral during a two-year period (2014 and 2015).

## 2. Materials and Methods

All hematopathology cases that were sent to AUB-MC for expert review between 2014 and 2015 were eligible to be included in the study. These included adult and pediatric cases. All nonhematopathology consult cases were excluded from the study. The cases were collected using the laboratory information system search engine. This retrospective study was approved by the Institutional Review Board at AUB-MC (IRB ID: PALM.SN.03).

All the referred cases were evaluated and interpreted by at least one specialized hematopathologist (SN or ZC). For every case, the pathology report issued at the referring institution and the report issued at AUB-MC were reviewed and the following information was extracted: patient's age and sex, country of origin of the referred material, type of specimen (excision versus needle-core biopsy), ancillary studies (if any) performed at the referring institution, ancillary studies (if any) performed at AUB-MC, outside diagnosis issued by the pathologist at the referring laboratory, and the final diagnosis issued by the hematopathologist at AUB-MC. Concordance was defined as having the same final pathologic diagnosis at the referring center and AUB-MC, while discordance was defined as having different final pathologic diagnoses between the two centers.

After review, the referred cases were divided into six main categories: 1- cases with discordant referral and postreview final diagnoses (discordant final diagnoses), 2- cases with discordant provisional referral diagnoses with immunohistochemical (IHC) studies recommended and postreview final diagnoses (discordant provisional diagnoses), 3- cases with concordant referral and postreview final diagnoses (concordant final diagnoses), 4- cases with concordant referral provisional diagnoses with IHC studies recommended and postreview diagnoses (concordant provisional diagnoses), 5- cases with vague (i.e., noncommitted) referral diagnoses and IHC studies recommended (vague diagnoses), and 6- cases where the received material was insufficient for diagnosis (insufficient for diagnosis). Some of the cases referred to more than one institution prior to presenting to AUB-MC and therefore multiple pathology reports were present for a single patient. In such instances, the case was grouped under the discordant category if at least one of the reports had a discrepant diagnosis, as this erroneous diagnosis potentially might have resulted in patients receiving incorrect treatment protocols. Furthermore, cases in category 2 were counted as discordant if patients were erroneously labeled as having a particular disease entity and committed to the corresponding workup protocol prior to review of the case, or in cases where a major discrepancy could be noted following examination of the hematoxylin and eosin (H&E) stained slides only (i.e., without ancillary testing). Additionally, in order to assess the potential therapeutic implications of discordant cases, discrepant diagnoses were divided into major and minor categories. Major discrepancies were defined as those that would significantly alter patient management while minor discrepancies were those that would not result in a significant change in patient care [12]. Diagnoses with major discrepancies were further subclassified into five categories: 1- nonhematologic malignancy reclassified as hematologic malignancy, 2- hematologic malignancy reclassified as nonhematologic malignancy, 3- benign hematologic diagnosis reclassified as malignant, 4- malignant hematologic diagnosis reclassified as benign, and 5- discrepancy in the classification of a hematologic malignancy.

After classification of the referred cases as per the above scheme, concordant and discordant rates were estimated. Also, cases with discordant diagnoses were further reviewed in an attempt to identify common and recurring sources or patterns of error that resulted in rendering erroneous diagnoses.

## 3. Results

**Figure 1:**
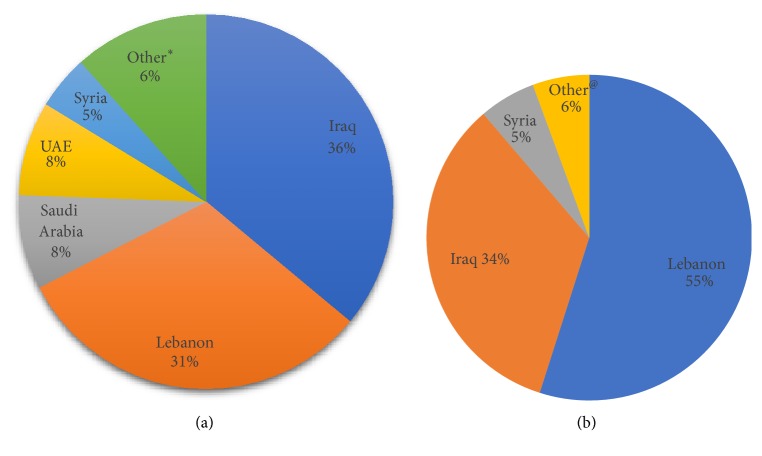
(a) Distribution of total referred cases per country. (b) Distribution of discrepant cases per country.^#^ (^#^Rates reported as percentages out of the total number of cases with discrepant diagnoses. *∗*Various countries including United States of America, Greece, Sweden, Jordan, Iran, and Yemen, with rare cases per country. ^@^Various countries including Saudi Arabia, Iran, Sweden, and United Arab Emirates.)

A total of 309 cases were collected. Specimens were received from a total of eighty-six centers in thirteen different countries and in variable proportions. The two major contributors were Iraq (36% of total cases) and from within Lebanon (31% of total cases), with smaller contributions from other countries including Saudi Arabia, the United Arab Emirates, and Syria (Figure 1(a)). Of note, cases from most of these countries included native patients as well as Lebanese patients living abroad and who sought review of their pathology material and potential treatment at AUB-MC. Reasons for referral ranged from lack of necessary ancillary testing in the referring laboratory, to challenging and unusual cases, to patients seeking second opinions, irrespective of the country of origin.

Of the total cases, seventy-one (23%) had major discrepancy in their diagnoses, and these included cases where ancillary testing was performed at the referring institution as well as cases where a provisional diagnosis was rendered solely based on examination of H&E-stained slides or with a limited immunohistochemical panel. The majority of the discrepant cases were from Lebanon (55%) and Iraq (34%). The three highest discrepancy rates per country were seen in cases referred from Lebanon (33%), followed by those from Syria (26%) and from Iraq (19%) (Figure 1(b) and Table 1).

When divided into diagnostic categories, 42% of all referred cases had concordant final diagnoses (category 3) (Table 2), 36% of the cases were signed out with a vague or provisional diagnosis (categories 2, 4, and 5), and 5% were insufficient for diagnosis with a repeat biopsy recommended (Table 2). Of the 54 cases with a discordant final diagnosis (category 1), 26 (48%) had their hematopoietic neoplasm reclassified into a different WHO-defined subtype (Table 3). The remaining 28 cases had major diagnostic revisions: 18 cases with benign diagnoses reclassified as malignant or vice versa and 10 cases initially considered as a nonhematologic malignancy were reclassified as a hematologic neoplasm and vice versa (Table 3). Benign/reactive conditions, nodular lymphocyte predominant Hodgkin lymphoma (NLPHL), and diffuse large B-cell lymphoma (DLBCL) constituted 56% of the cases with discrepant diagnoses (Table 4). The majority of cases of NLPHL that were misdiagnosed had a referral diagnosis of classical Hodgkin lymphoma (CHL) (11 of 13 cases; 85%), and 4 of the 5 cases of CHL that had a discrepant diagnosis were diagnosed outside as reactive changes (Table 4).

Of note, when considering the referrals with provisional diagnoses overall, those with concordant diagnoses included examples of follicular lymphoma, CHL, plasma cell myeloma, extranodal marginal zone lymphoma of mucosa-associated lymphoid tissue (MALT lymphoma), and bone marrows and lymph nodes with no evidence of malignancy. Cases with discordant provisional diagnoses included cases of NLPHL misdiagnosed as CHL, reactive conditions misdiagnosed as malignant (e.g., chronic gastritis as MALT lymphoma, infectious mononucleosis as large cell lymphoma, reactive lymphadenitis as T-cell lymphoma based on the interfollicular expansion), a lymphoma misdiagnosed as a nonhematopoietic tumor (MALT lymphoma as hemangiopericytoma), and a nonhematopoietic tumor misdiagnosed as lymphoma (inflammatory myofibroblastic tumor as CHL).

## 4. Discussion

The diagnosis of hematopoietic neoplasms remains very challenging. The ongoing classification changes and the rapid evolution of our understanding only added more difficulty to the challenge. Discrepancy rates varied significantly in previous studies done in various countries, as was shown in studies done in Taiwan (55%) [13], Turkey (45.6%) [15], East Netherlands (9%) [14], France (19.7%) [16], and the United Kingdom (27.3%) [11]. In the United States of America, the rate of major diagnostic discrepancies was in different studies 6% [1], 14.8% [12], and 18.6% [7]. In our study, the overall percentage of discrepant cases for which a final or provisional diagnosis was made was 23%.

Several difficulties were identified while reviewing the consult cases, which likely contributed as sources of error precluding accurate initial diagnoses. Many of the received cases did not have sufficient corresponding clinicoradiologic information, and the histologic findings were not placed in the proper clinical contexts. In some instances, even after expert review, there were comments in the final reports emphasizing the importance of clinicopathologic correlation findings, as definitive diagnoses could not be reached with the provided clinical information. Lack of patient clinical data was particularly hindering in situations with limited tissue availability, such as small needle-core biopsies with near tissue depletion after immune-histochemistry was attempted (5% of cases, Table 3). In such situations, it might have been more prudent to issue reports with descriptive diagnoses and recommend examination of additional material and clinicoradiologic correlation rather than committing to a specific diagnosis. Another problem encountered was the limited quality of the H&E-stained sections received from several of the referring laboratories. Improper H&E evaluation may lead to erroneous final or provisional diagnoses, delays in reaching a final diagnosis (by ordering unnecessary ancillary testing), and increased total costs for the patients. Furthermore, in laboratories where immunohistochemical studies are not available, an improper morphologic assessment could result in an inaccurate provisional diagnosis which may dramatically affect the immediate management of the patient. Review of the cases with discordant diagnoses based on H&E-stained slide evaluation alone (6% of cases, Table 2) showed essentially sections with limited preservation of morphology due to various causes including poor fixation/ inadequate tissue processing, thick sections, and extensive tissue folding while sectioning the paraffin blocks. Unfortunately, immunohistochemical studies, an integral part of the evaluation of hematopathology cases, were not available in many of the referring laboratories, which lead to provisional and ambiguous diagnoses being rendered in around 36% of the total referred cases. Lack of immunohistochemical studies combined with limited H&E-stained slide quality often resulted in management delay as these cases were referred to other laboratories where immunohistochemical studies were available.

The aforementioned causes for discrepancies were mostly noted in cases referred from Lebanon, Syria, and Iraq. However, specific conclusions regarding the reasons behind this finding could not be made without collecting data directly from referring pathologists in these countries (a task beyond the scope of this study). Additionally, many of the consult cases with these issues were received with reports or clinical notes not clearly identifying the referral laboratory. Therefore, it was difficult to determine the number of cases that originated from different laboratories versus those that originated from the same laboratory but were examined by different pathologists.

**Figure 2:**
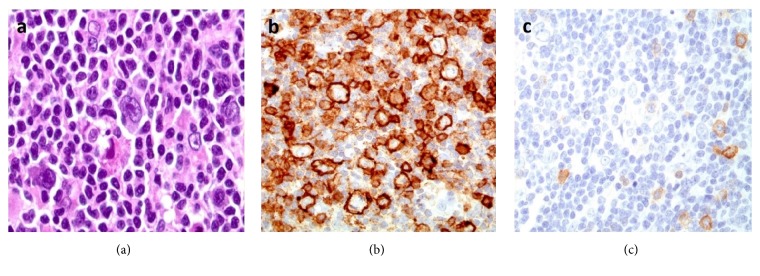
Nodular lymphocyte predominant Hodgkin lymphoma. (a) Lymphocyte predominant (LP) cells with folded and multilobated nuclei. (b) The LP cells are positive for CD20. (c) CD30 is positive in scattered immunoblasts but negative in the LP cells. This case was misclassified as classical Hodgkin lymphoma.

Interestingly, nodular lymphocyte predominant Hodgkin lymphoma (NLPHL) was the single most frequently misdiagnosed entity, accounting for 13% of the cases with a discrepant final or provisional diagnosis (Table 4), and it was most commonly misdiagnosed as classical Hodgkin lymphoma (CHL) in 11 of 13 cases (Table 4). We noticed two patterns of error leading to such a misdiagnosis. In a subset of cases, there seemed to be a lack of awareness of NLPHL, such that it was often not included in the differential diagnoses in cases where Reed Sternberg- (RS-) like cells were found, despite the presence of typical histologic patterns. Another source of error was overinterpretation of the CD30 immunohistochemical stain. In several cases of NLPHL, CD30 staining was seen in bystander immunoblasts, and these were confused with tumor cells by the examining pathologist (Figure 2). Of note, there were examples of benign reactive conditions misdiagnosed as CHL for the same reason. Furthermore, weak and partial CD30 positivity can be seen in the neoplastic cells of NLPHL, and such a finding by itself is not sufficient for a diagnosis of CHL without the other typical immunophenotypic features of this neoplasm [9].

Diffuse large B-cell lymphoma (DLBCL), as expected, was one of the most common non-Hodgkin lymphomas and was one of the most common diagnoses among the referred cases. While rarely misdiagnosed, many of the DLBCL cases diagnosed outside were not classified into germinal center B-cell-like (GCB) or activated B-cell-like (ABC) subtypes. The 2008 edition of the WHO recognized and emphasized the GCB and ABC subtypes of DLBCL, and the 2016 update recommended the identification of these subtypes either by gene expression profiling or through immunohistochemistry algorithms [8, 9, 17] for prognostic purposes. In addition, most of the referred cases of large B-cell lymphomas were diagnosed outside without testing for certain markers that help further classify this neoplasm, as specific subtyping was shown to carry a clinical significance. Such workup, also recommended by the WHO 2016 update, should include testing for EBV [9, 17] and FISH studies for C-MYC and (if needed) BCL-2 and BCL-6 gene rearrangements [9, 17, 18]. Testing for C-MYC/BCL-2/BCL-6 mutations was recommended particularly for cases with high-grade features and aggressive clinical behavior. While not being sources of misdiagnosis by themselves, these findings further highlight a deficiency in the general awareness of important updates in the field of hematopathology.

**Figure 3:**
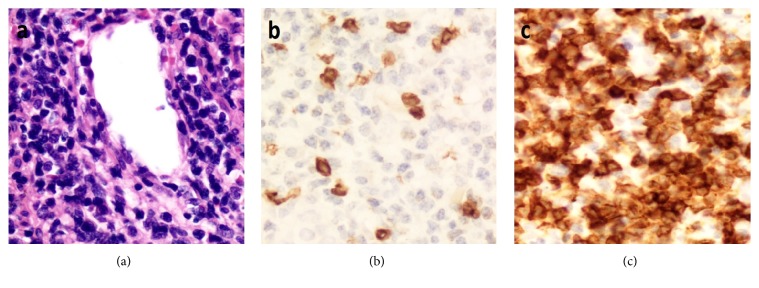
Extranodal NK/T-cell lymphoma, nasal type. (a) The lymphoma cells are small to medium-sized and are angioinvasive. There is significant downregulation of CD3 (b) when compared to CD5 (c). The tumor cells are also positive for EBER and CD56 (not shown).

**Figure 4:**
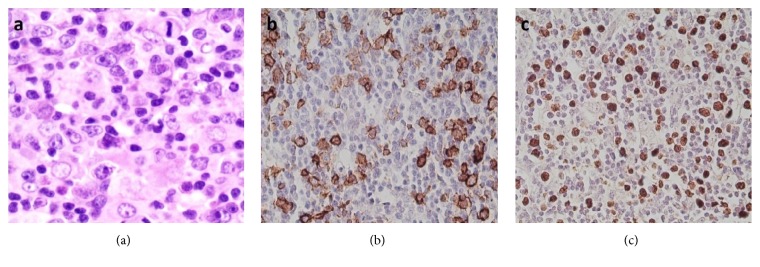
Infectious mononucleosis. A florid proliferation of immunoblasts can be seen (a), which are positive for CD20 (b) and EBER (c) and may be confused with diffuse large B-cell lymphoma.

Of note, of the 54 cases with a discrepant final diagnosis, 28 (52%) had either a benign diagnosis that was reclassified as lymphoma (and vice versa) or had a nonhematological diagnosis that was reclassified as hematological (and vice versa) (Table 3). Such major revision of the diagnoses can have a major impact on the management and the outcome of patients. One such case belonged to a patient who presented with a mass in the nasal cavity diagnosed at an outside laboratory as an inflammatory granulation tissue polyp after immunohistochemical studies were done. After review, this case was reclassified as extranodal NK/T-cell lymphoma, nasal type, where a significant downregulation of a pan T-cell marker was missed by the referring pathologists, in addition to several histologic findings that supported a diagnosis of malignancy (Figure 3) [9, 19]. Failure to detect these aberrancies precluding testing for additional useful markers including CD56 and EBER, resulted in an erroneous diagnosis of a reactive process. The value of this case was that it highlighted several clues to the diagnosis, including the importance of assessing and comparing multiple pan T-cell markers to detect T-cell aberrancies. However, it should be noted that subtle T-cell aberrancies should always be placed in the proper histologic context, as was shown in a case of a 43-year-old patient who presented with fever, cervical lymphadenopathy, and splenomegaly. The patient was diagnosed at an outside laboratory as having a peripheral T-cell lymphoma, supported by the downregulation of CD5 and CD7 in T-cells. After review of the pathology material and after clinical correlation was made, a diagnosis of acute infectious mononucleosis (IM) was rendered, with the knowledge that T-cell antigenic aberrancies can occur in cases of IM [20, 21], and the overall histologic and immunohistochemical findings that were more in keeping with an inflammatory process than a neoplasm. The review diagnosis was further confirmed by the patient's follow-up clinical course (resolution of symptoms and lymphadenopathy without chemotherapy) and by serologic testing which confirmed the presence of EBV IgM antibodies. Other EBV-related reactive changes can be misleading [21]. For example, another case of IM showed florid proliferation of immunoblasts, which lead to the erroneous diagnosis of diffuse large B-cell lymphoma (Figure 4). Additionally, there was a case of chronic active EBV infection in a pediatric patient where Reed-Sternberg-like cells were present, resulting in an incorrect diagnosis of Hodgkin lymphoma. The diagnosis of IM/EBV infection in lymph nodes, though not very common, can be very difficult at times with many potential pitfalls. This fact stresses on the importance of integration of morphologic, immunohistochemical, and clinical information, in order not to overdiagnose EBV-associated inflammatory conditions as hematopoietic neoplasms.

**Figure 5:**
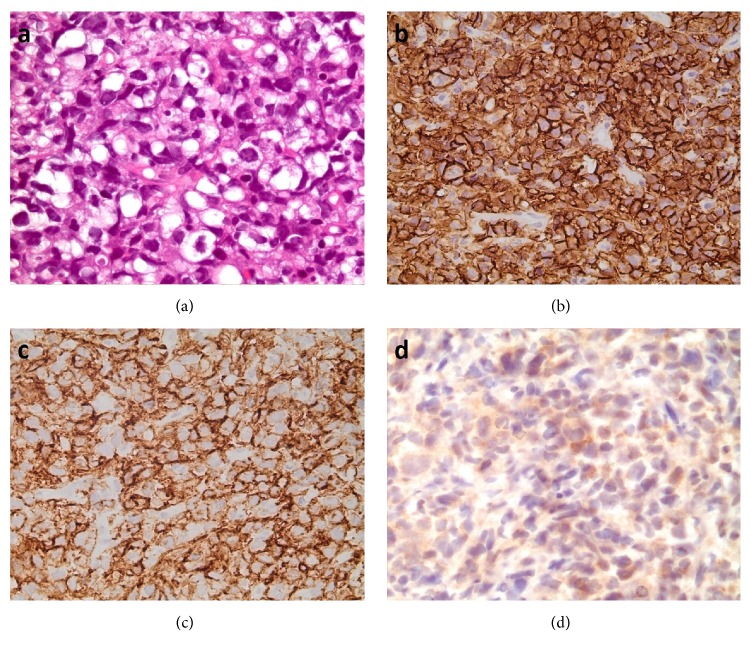
Primary mediastinal (thymic) large B-cell lymphoma. (a) The tumor cells are large with pleomorphic nuclei and abundant vacuolated cytoplasm. They are positive for CD20 (b) and CD23 (c) and show nonspecific cytoplasmic staining with TdT (d). This case was misclassified as a B-lymphoblastic leukemia/lymphoma.

**Figure 6:**
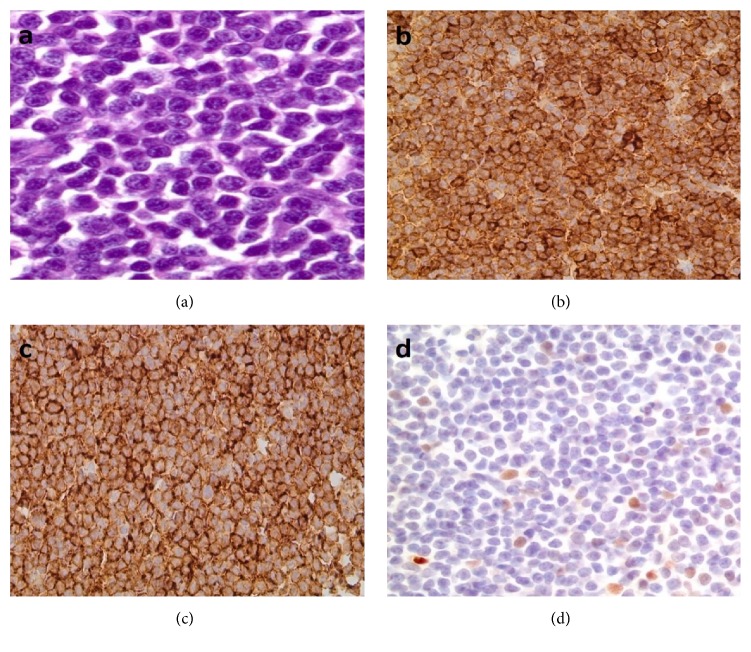
Small lymphocytic lymphoma/chronic lymphocytic leukemia. (a) High magnification of a proliferation center composed predominantly of prolymphocytes and paraimmunoblasts. The lymphoma cells are positive for CD5 (b) and CD23 (c). (d) Cyclin-D1 showed positivity in scattered cells.

Lastly, two interesting patterns of error were noted in the interpretation of two immunohistochemical nuclear stains. The first pattern related to the misinterpretation of the TdT immunohistochemical stain, where two referred cases were misdiagnosed as lymphoblastic leukemia/lymphoma based on TdT positivity. Upon review of these cases, it was noted that both cases showed nonspecific cytoplasmic staining rather than the true nuclear staining typically seen with TdT. One case initially diagnosed as B-lymphoblastic leukemia/lymphoma (B-LBL) was reclassified as a primary mediastinal (thymic) large B-cell lymphoma (Figure 5). Another case was originally diagnosed as T-lymphoblastic leukemia/lymphoma based on erroneous TdT interpretation and CD56 positivity in the tumor cells, but was reclassified as a rhabdomyosarcoma as a repeat TdT stain in our laboratory was negative, and the tumor cells were diffusely and strongly positive for muscle markers. The second pattern of error related to the interpretation of the cyclin-D1 immunohistochemical stain. Two cases were referred to our institution for a bone marrow transplant due to a diagnosis of mantle cell lymphoma. Review of both cases showed findings consistent with chronic lymphocytic leukemia/small lymphocytic lymphoma (CLL/SLL), and both cases had scattered cyclin-D1 positive cells predominantly within proliferation centers, rather than the diffuse staining typically seen in cases of mantle cell lymphoma (Figure 6). Focal cyclin-D1 expression is known to occur in up to 30% of cases of CLL/SLL [9], and this finding should be recognized in order to avoid misdiagnosing such cases as mantle cell lymphoma, which may drastically alter the course of patient management. It is important to note that one problem that several of the referring laboratories faced was the unavailability of the cyclin-D1 immunohistochemical stain. As a result, several referring pathologists were unable to confidently diagnose mantle cell lymphoma in the absence of molecular/in situ hybridization studies.

## 5. Conclusions

In conclusion, the high rate of major diagnostic revision observed in our study emphasizes the importance of referring hematopathology cases for expert review, in particular when the general pathologist is uncertain of the diagnosis or when immunohistochemical stains are not sufficiently available. Furthermore, when a vague or ambiguous diagnosis is issued, it is important for the clinician not to start treatment until referral to a more specialized center occurs and a more specific diagnosis is rendered. The present study, despite its limitation (small number of consult cases reviewed), highlighted some of the major difficulties that pathologists in Lebanon and in the Middle East face and shed some light on some of the patterns of diagnostic errors observed, in an attempt to improve some aspects of the practice of hematopathology in the region.

## Figures and Tables

**Table 1:** Discrepancy rates per country for Lebanon, Syria, and Iraq.

Country of origin	Total cases received per country	Number of discrepant cases per country (%)
Lebanon	95	31 (33)

Syria	15	4 (26)

Iraq	100	19 (19)

**Table 2:** Distribution of total referred cases per diagnostic category.

Major diagnostic categories	Total number per category (%)
Discordant final diagnoses (category 1)	54 (17)

Discordant provisional diagnoses (category 2)	17 (6)

Concordant final diagnoses (category 3)	131 (42)

Concordant provisional diagnoses (category 4)	34 (11)

Vague diagnoses (category 5)	59 (19)

Insufficient for diagnosis (category 6)	14 (5)

Total number of referred cases	309 (100)

**Table 3:** Distribution of cases with discrepant final diagnoses.

Diagnostic revision categories	Total number per category (%)
Discrepancy in subtype classification	26 (48)

Benign diagnosis reclassified as malignant	9 (17)

Malignant diagnosis reclassified as benign	9 (17)

Non-hematologic malignancy reclassified as hematologic malignancy	7 (13)

Hematologic malignancy reclassified as non-hematologic malignancy	3 (5)

Total number of cases	54 (100)

**Table 4:** List of discrepant cases^*∗*^ with referral and postreview diagnoses.

Diagnosis after review (number of cases with a given diagnosis)	Referral diagnosis (number of cases; %)
Benign/reactive/inflammatory findings (16)	Marginal zone lymphoma/MALT lymphoma (10; 63) T-cell lymphoma (3; 19) Large cell lymphoma, not further specified (1; 6) Classical Hodgkin lymphoma (1; 6) Langerhans histiocytosis (1; 6)

Nodular lymphocyte predominant Hodgkin lymphoma (13)	Classical Hodgkin lymphoma (11; 85) DLBCL (2; 15)

Diffuse large B cell lymphoma (11)	Carcinoma (4; 37) Classical Hodgkin (2; 18) MALT lymphoma (1; 9) Burkitt lymphoma (1; 9) Follicular lymphoma (1; 9) Germinoma (1; 9) Osteogenic sarcoma (1; 9)

Classical Hodgkin lymphoma (5)	Reactive changes (4; 80) Granulomatous inflammation (1; 20)

Follicular lymphoma, low grade (4)	Reactive changes (2; 50) Small lymphocytic lymphoma, diffuse (1; 25) High grade follicular lymphoma (1; 25)

CLL/SLL (4)	Mantle cell lymphoma (2; 50) High grade lymphoma (1; 25) Malignant myeloid infiltrate (1; 25)

Possible double-hit lymphoma (2)	Burkitt (2; 100)

MALT lymphoma (2)	Diffuse large B-cell lymphoma (1; 50) Hemangiopericytoma (1; 50)

Extranodal NK/T cell lymphoma, nasal type (2)	Peripheral T-cell lymphoma, NOS (1; 50) Reactive changes (1; 50)

Peripheral T-cell lymphoma, NOS (2)	CLL/SLL (1; 50) Carcinoma (1; 50)

Mantle cell lymphoma (1)	Small lymphocytic lymphoma (1; 100)

PMLBCL (1)	B-lymphoblastic lymphoma (1; 100)

EBV+ large B-cell lymphoma (1)	Classical Hodgkin lymphoma (1; 100)

Acute myeloid leukemia (1)	Chronic myelomonocytic leukemia (1; 100)

Rhabdomyosarcoma (1)	T-lymphoblastic lymphoma (1; 100)

Metastatic carcinoma (1)	Multiple myeloma (1; 100)

Thymoma (1)	MALT lymphoma (1; 100)

Atypical lymphoid proliferation, not further classified^@^ (1)	Reactive changes (1; 100)

Aplastic anemia (1)	Normocellular bone marrow (1; 100)

Inflammatory myofibroblastic tumor (1)	Classical Hodgkin lymphoma (1; 100)

^*∗*^The discrepant cases include those with a referral final or provisional diagnosis.

^@^ The received tissue was insufficient for further evaluation, but the findings on the H&E slides were compatible with a lymphoma.

## Data Availability

The data used to support the findings of this study are restricted by the Institutional Review Board at the American University of Beirut in order to protect patient privacy.
